# A220 ONTARIO POPULATION TRENDS IN INTESTINAL AND EXTRA-INTESTINAL CANCERS OVER 25 YEARS AMONG PERSONS WITH INFLAMMATORY BOWEL DISEASES AND MATCHED CONTROLS

**DOI:** 10.1093/jcag/gwac036.220

**Published:** 2023-03-07

**Authors:** S K Murthy, G G Kaplan, S Coward, E Kuenzig, E I Benchimol, A Zubieta, A Otley, A Bitton, C N Bernstein, L Targownik, J Jones, J Begum, M Pugliese, H Singh

**Affiliations:** 1 Medicine, University of Ottawa, Ottawa; 2 Medicine, University of Calgary, Calgary; 3 Pediatrics, University of Toronto, Toronto; 4 Medicine, University of British Columbia, Vancouver; 5 Pediatrics, Dalhousie University, Halifax; 6 Medicine, McGill University, Montreal; 7 Medicine, University of Manitoba, Winnipeg; 8 Medicine, University of Toronto, Toronto; 9 Medicine, Dalhousie University, Halifax; 10 Institute for Clinical Evaluative Sciences, Ottawa , Canada

## Abstract

NOT PUBLISHED AT AUTHOR’S REQUEST

**Please acknowledge all funding agencies by checking the applicable boxes below:**

CCC, CIHR

**Disclosure of Interest:**

S. Murthy: None Declared, G. Kaplan Grant / Research support from: Ferring, Janssen, AbbVie, GlaxoSmith Kline, Merck, and Shire, Consultant of: AbbVie, Janssen, Pfizer, Amgen, Takeda, Gilead, Speakers bureau of: AbbVie, Janssen, Pfizer, Amgen, and Takeda, S. Coward: None Declared, E. Kuenzig: None Declared, E. Benchimol Consultant of: Hoffman La-Roche Limited, Peabody & Arnold LLP, McKesson Canada, Dairy Farmers of Ontario for matters unrelated to medications used to treat inflammatory bowel disease., A. Zubieta: None Declared, A. Otley: None Declared, A. Bitton: None Declared, C. Bernstein Grant / Research support from: Abbvie Canada, Janssen Canada, Pfizer Canada, Bristol Myers Squibb Canada, Takeda Canada; Amgen Canada, Sandoz Canada , Consultant of: Abbvie Canada, Amgen Canada, Bristol Myers Squibb Canada, JAMP Pharmaceuticals, Janssen Canada, Pfizer Canada, Sandoz Canada, Takeda, Speakers bureau of: Abbvie Canada, Janssen Canada, Pfizer Canada and Takeda Canada, L. Targownik Grant / Research support from: Janssen Canada, Consultant of: AbbVie Canada, Takeda Canada, Merck Canada, Pfizer Canada, Janssen Canada, Roche Canada, and Sandoz Canada, J. Jones Consultant of: Janssen, Abbvie, Pfizer, Takeda, Speakers bureau of: Janssen, Abbvie, Pfizer, Takeda, J. Begum: None Declared, M. Pugliese: None Declared, H. Singh Consultant of: Pendopharm, Amgen Canada, Bristol Myers Squibb Canada, Roche Canada, Sandoz Canada, Takeda Canada, and Guardant Health, Inc.,

